# Foveal structure and vasculature in eyes with idiopathic epiretinal membrane

**DOI:** 10.1371/journal.pone.0214881

**Published:** 2019-04-02

**Authors:** Yuka Okawa, Ichiro Maruko, Moeko Kawai, Taiji Hasegawa, Hisaya Arakawa, Tomohiro Iida

**Affiliations:** Department of Ophthalmology, Tokyo Women’s Medical University, Shinjuku, Tokyo, Japan; Sankara Nethralya, Medical Research Foundation, INDIA

## Abstract

**Purpose:**

To examine the foveal structure and vasculature in eyes with an idiopathic epiretinal membrane (ERM).

**Methods:**

Forty-nine eyes of 48 patients with an idiopathic ERM were studied. The superficial foveal avascular zone (FAZ) was measured by optical coherence tomography angiography (OCTA; RTVue XR Avanti, Optovue Inc., Fremont, CA), and the central foveal thickness (CFT) was measured by swept source OCT (DRI-OCT, Topcon, Japan). Twenty eyes underwent vitrectomy with internal limiting membrane (ILM) peeling, and the FAZ and CFT were evaluated pre- and postoperatively. Forty-nine eyes of 49 age-matched healthy subjects were also examined as control.

**Results:**

The FAZ in eyes with an ERM was significantly smaller than that of the control eyes (0.188±0.16 mm^2^ vs 0.328±0.14 mm^2^, *P*<0.01). The CFT in eyes with an ERM was significantly thicker than that of control eyes (315±0.14 μm vs 193±0.14 μm, *P*<0.01). The size of the FAZ was strongly correlated with the CFT (ERM, R = -0.753; control, R = -0.61, both *P*<0.01). The postoperative size of the FAZ was not significantly different from the preoperative size (0.115 mm^2^ vs 0.128 mm^2^, *P* = 0.17) but the CFT was significantly thinner (370 μm vs 288 μm, *P*<0.01) after the vitrectomy with ILM peeling in 20 eyes.

**Conclusions:**

The results indicate that an ERM might affect the morphology and vasculature of not only the inner but also the outer retina before and after vitrectomy with ILM peeling. The FAZ area might have been affected by the ILM peeling.

## Introduction

An idiopathic epiretinal membrane (ERM) can alter the foveal morphology by its traction on the retina, and the morphological changes can cause distortions of vision and reductions of the visual acuity. The results of optical coherence tomographic (OCT) studies have shown a thickening of the fovea and a disruption of the ellipsoid zone of the photoreceptors in eyes with an ERM [1–5]. The disruption of the ellipsoid zone indicates a disorder in the alignment of the photoreceptors which most likely accounts for the visual dysfunctions. Inoue et al [4] reported that the improvement of the visual acuity after removing an ERM was not significant after vitreous surgery. However, because there is no inner retina at the fovea, these reports only evaluated the changes of the outer retina in eyes with an idiopathic ERM.

There are recent studies that report that the visual abnormalities can be present in eyes with good visual acuities and without any disruption of the ellipsoid zone. This was suggested to result from changes in the inner retina caused by the ERM [6–8].

However, it has not been adequately reported that the inner retina is altered in eyes with an ERM.

Thus, the purpose of this study was to determine the effect of an ERM on the inner retina. To accomplish this, the superficial foveal avascular zone (FAZ) was measured by optical coherence tomography angiography (OCTA), and the central foveal thickness (CFT) was measured by swept source OCT in eyes with an ERM. In addition, the areas of the FAZ and CFT were evaluated after vitrectomy with ERM and internal limiting membrane (ILM) peeling.

## Materials and methods

This was a retrospective study, and the procedures used conformed to the tenets of the Declaration of Helsinki. The Institutional Review Board of the Tokyo Women’s Medical University, School of Medicine approved the procedures used. All examinations were performed after an informed consent was obtained.

Forty-nine eyes of 48 patients (25 men, 24 women; average age, 67.1 years) with an idiopathic ERM were examined by OCTA (RTVue XR Avanti, Optovue Inc., Fremont, CA) with a scan of 3 x 3 mm centered on the fovea. Eyes with high myopia, diabetic retinopathy, retinal vein occlusion, a history of pars plana vitrectomy, and inflammation other than secondary to an ERM were excluded. Eyes without a posterior vitreous detachment were also excluded to avoid cases of secondary ERM. In 20 eyes among all eyes, the FAZ was evaluated after pars plana vitrectomy with ERM and ILM peeling. Cataract surgery was performed at the time of vitrectomy in all eyes. Forty-nine eyes of 49 age-matched healthy individuals were also examined as controls. None of these eyes had an ERM and had undergone vitrectomy.

The OCTA (RTVue XR Avanti) instrument can record the microvascular structures using a split spectrum amplitude-decorrelation angiography without fluorescein or indocyanine green dye. This device records 70,000 A-scans/second with motion correction which helps in recording clear *en face* OCTA images. The software embedded in the OCTA device automatically shows four *en face* OCTA images divided into four depths; the superficial capillary plexus, deep capillary plexus, outer retina, and choriocapillaris layers. In this study, the superficial capillary plexus was used to avoid segmentation errors. Eyes with blurred OCTA images were excluded even if the motion correction technology was used.

The area of the FAZ was measured by the polygon selection tool of the ImageJ software (National Institutes of Health, Bethesda, MD; available at http://rsb.info.nih.gov/ij/index.html). The measurement of the FAZ area was done for only the superficial layer images because the FAZ area in the deep plexus layer can be affected by projection artifacts. All FAZ areas were measured by two co-authors (YO and IM), and their values were averaged. The change in the ratio of FAZ area after vitrectomy was calculated as, (postoperative FAZ area–preoperative FAZ area)/preoperative FAZ area.

All eyes were also examined by swept-source OCT (DRI-OCT, Topcon, Japan), and the images were used to measure the central foveal thickness (CFT). The CFT was determined with the caliper tool in the OCT software. The change in the ratio of CFT after vitrectomy was calculated as, (postoperative CFT–preoperative CFT)/preoperative CFT.

We also evaluated the irregularity and/or disruption of the ellipsoid zone of the photoreceptors in the cross-sectional images. Twenty eyes underwent vitrectomy with ERM and ILM peeling, and we determined whether the foveal contour was restored in the DRI-OCT images. We classified eyes as having a restored foveal contour when the retinal thickness at the center of the fovea was at least 50 μm thinner than that of the retina 1 mm away from the foveola without intraretinal edema [9].

The BCVA was measured with a Japanese standard decimal visual chart, and the decimal visual acuities were converted to the logarithm of the minimum angle of resolution (logMAR) for the statistical analyses.

### Statistical analyses

Mann–Whitney U-tests were used to determine the significance of the differences in the FAZ area and CFT between the ERM and control groups. Wilcoxon signed-rank tests were used to determine the significance of the differences in the FAZ area, CFT, and BCVA before and after the ERM surgery. Spearman’s tests were used to determine the significance of the correlations. All *P*-values were two-sided and values <0.05 were considered statically significant. All statistical analyses were performed with free software EZR (Saitama Medical Center, Jichi Medical University, Saitama, Japan), which is a graphical user interface for R (The R Foundation for Statistical Computing, Vienna, Austria) [10]. More exactly, it is a modified version of the R commander designed to add statistical functions frequently used in biostatistics.

## Results

**Fig 1:**
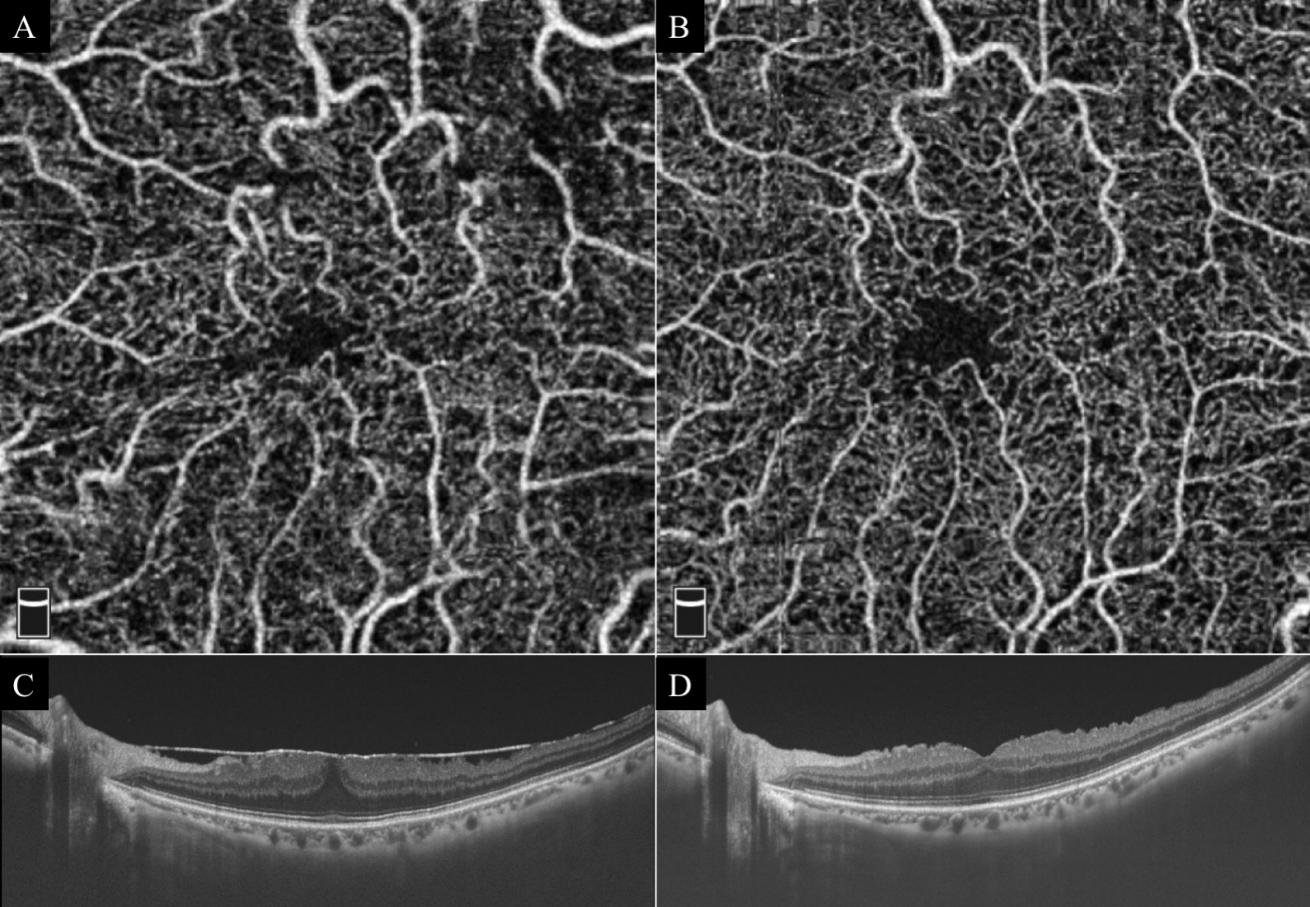
Representative case with an epiretinal membrane (EM). The foveal avascular zone (FAZ) increased and the central foveal thickness (CFT) decreased after vitrectomy with peeling of the internal limiting membrane (ILM). **A:** Superficial optical coherence tomography angiography (OCTA) image before vitrectomy. Area of FAZ is 0.072 mm^2^. **B:** Superficial OCTA image after vitrectomy. Area of FAZ is 0.148 mm^2^. **C:** Cross-sectional OCT image before vitrectomy. The central foveal thickness (CFT) is 405 μm. **D:** Cross-sectional OCT image after vitrectomy. The CFT is 270 μm.

**Fig 2:**
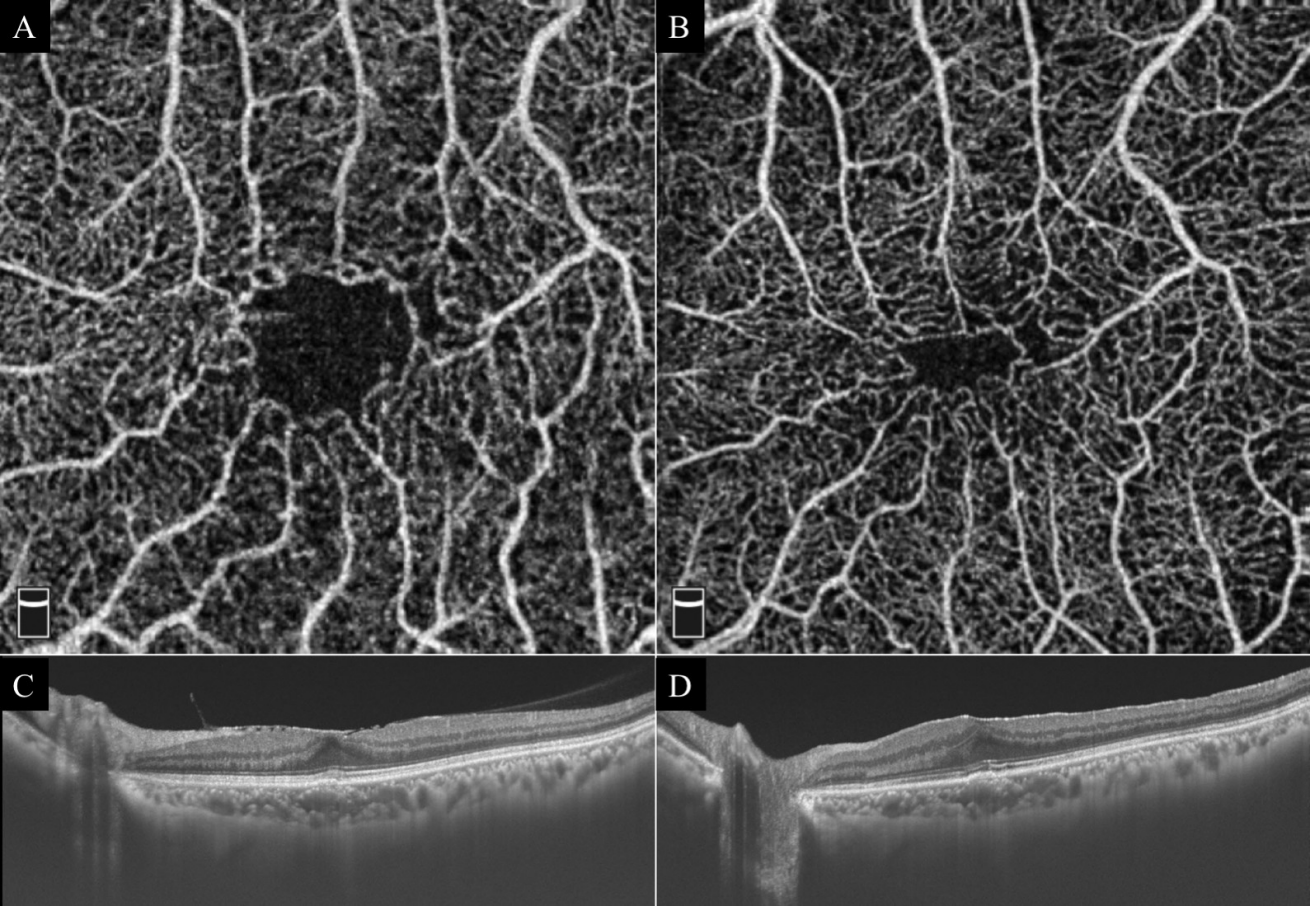
Representative case with a reduction of the FAZ and an increase in the CFT after vitrectomy with ILM peeling. **A:** Superficial OCTA image before vitrectomy. Area of FAZ is 0.426 mm^2^. **B:** Superficial OCTA image after vitrectomy. Area of FAZ is 0.119 mm^2^. **C:** Cross-sectional OCT image before vitrectomy. CFT is 280 μm. **D:** Cross-sectional OCT image after vitrectomy. CFT is 335 μm.

The mean area of the FAZ was 0.188 ± 0.16 mm^2^ in the eyes with an ERM which was significantly smaller than the 0.328 ± 0.14 mm^2^ in the control eyes (*P* <0.01). The FAZ area in the eyes with an ERM was not significantly correlated with the age (R = 0.048, *P* = 0.74) or the BCVA (R = -0.233, *P* = 0.11). The area of the FAZ increased from 0.115 mm^2^ to 0.128 mm^2^ after vitrectomy in the 20 eyes with a mean follow-up period of 147 days (range 23–443 days). This increase was not significant (*P* = 0.17). The mean postoperative FAZ area was significantly smaller than that of the control eyes (*P* <0.01). The FAZ area enlarged in 14 eyes (Fig 1) and decreased in 6 eyes (Fig 2). The mean area of the FAZ with enlargement after vitrectomy was smaller than decrease in the area of the FAZ in the other eyes (0.091 mm^2^ vs 0.171 mm^2^, *P* = 0.06). The mean change in the ratio was not significantly correlated with the age (R = 0.421, *P* = 0.06) and the length of the follow-up period (R = -0.357, *P* = 0.12). Seven eyes were followed for more than 6 months, and the FAZ area increased in 2 eyes and decreased in 5 eyes. Although there was no significant difference between pre- and postoperative FAZ areas (0.166 mm^2^ vs 0.11 mm^2^, *P* = 0.30) in these eyes, the change in the ratio of the areas in the eyes with more than 6 months of follow-up was significantly smaller than that of eyes with less than 6 months of follow-up (-0.170 vs 0.750, *P* <0.01).

The mean CFT in the eyes with an ERM was significantly thicker than that of the control eyes (315 ± 22 μm vs 193 ± 100 μm, *P* <0.01), and the CFT was significantly correlated with the BCVA (R = 0.315, *P* = 0.03) but not with the age (R = -0.102, *P* = 0.49). The CFT decreased significantly from 370 μm to 288 μm after vitrectomy in the 20 eyes (*P* <0.01), however the mean postoperative CFT was still significantly thicker than that of the control eyes (*P* <0.01). In the eyes that underwent vitrectomy and had an increase in the area of the FAZ, the CFT was significantly thicker than in the eyes with a reduction in the size of the FAZ (393 μm vs 316 μm, *P* <0.01). The mean change in the ratio of the areas was not correlated with the age (R = -0.046, *P* = 0.85) and the duration of the follow-up period (R = 0.371, *P* = 0.11).

**Fig 3:**
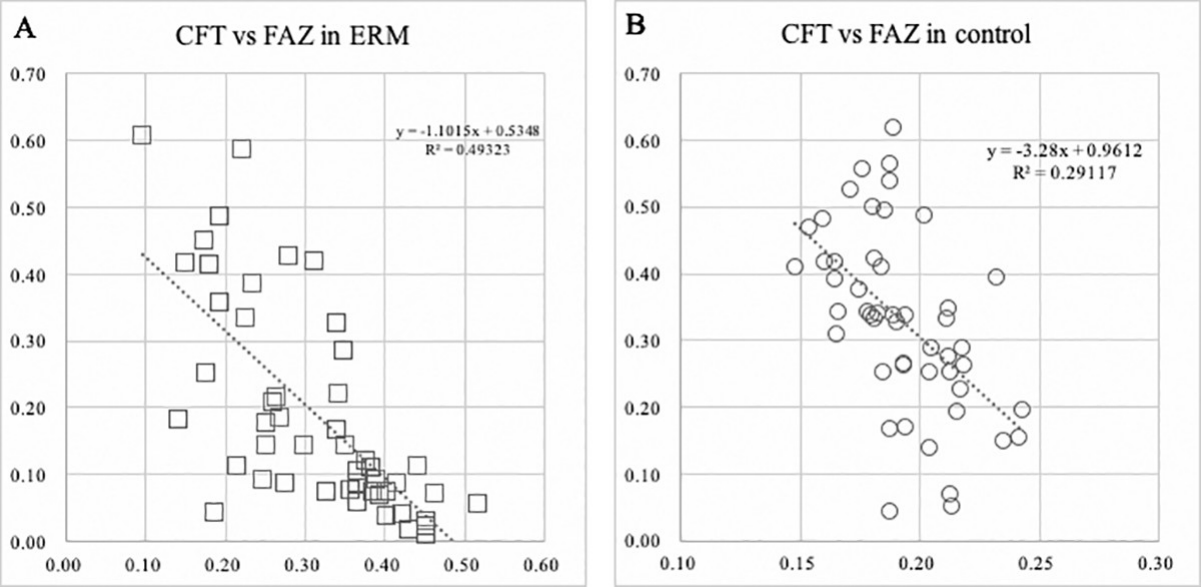
Plot showing the relationship between the FAZ area and the CFT. The FAZ is negatively and significantly correlated with the CFT in the eyes with an ERM (**A**), and control eyes (**B**).

The area of the FAZ was negatively and significantly correlated with the CFT in the eyes with an ERM (R = -0.753, *P* <0.01, Fig 3A) and also in the control eyes (R = -0.610, *P* <0.01, Fig 3B). The change in the ratios of both the FAZ area and the CFT were significantly correlated (R = -0.743, *P* <0.01).

There were no abnormalities of the ellipsoid zone at the fovea in the cross-sectional OCT images. The restoration of the foveal contour after vitrectomy was detected in 12 (63.2%) of 19 eyes without a foveal contour before the vitrectomy. An enlargement of the FAZ was found after vitrectomy in 10 (83.3%) of 12 eyes with the restoration of the foveal contour.

The mean decimal BCVA in all eyes with an ERM was 0.90, and that of the 20 eyes treated with vitrectomy was 0.88 at the baseline and 1.02 after the surgery. The BCVA after the vitrectomy was not significantly changed (*P* = 0.13).

The findings in all of the eyes with an ERM are summarized in Supplemental Table (S1 Table).

## Discussion

The results showed that the area of the FAZ was smaller and the CFT was thicker in the eyes with an ERM than in age-matched control eyes. This difference was still present after successful removal of the ERM by vitrectomy with ILM peeling. This indicated that the tractional effects of ERM might persist even after its removal. Although the CFT became significantly thinner after vitrectomy, the decrease in the FAZ area was not significant. These results may be due to not only the centrifugal effect of ERM removal but also the centripetal movement of the retina after the ILM peeling.

An ERM can cause distortions of vision and visual dysfunction through a traction on the surface of the retina. Thus, vitrectomy is required to release the traction. Since the early times of OCT [1–5], alterations of various parameters associated with the visual function were detected in eyes with an ERM, e.g., thicker CFT, greater distortion of vision, good preoperative vision, better postoperative vision, greater postoperative reduction of the CFT, better visual prognosis, changes in the postoperative foveal depression correlated with the postoperative vision, and the integrity of the ellipsoid zone that were significantly correlated with the postoperative vision. However, these studies mainly evaluated the changes of the outer retina because an inner retina did not exist at the fovea. In our cohort, none of the eyes had a disruption of the ellipsoid zone. Most recent studies focused on the inner retinal layer in eyes with an ERM and without any abnormalities of the outer retina. Koo et al [6] evaluated the inner retina from volume scans of eyes with an ERM, and they reported that the thickness of the inner retina at the parafovea (within 3 mm) was significantly associated with the visual dysfunction even with an intact photoreceptor layer. Joe et al [7] reported that the presence of an inner retinal layer at the fovea was the major determinant of the visual acuity in eyes with an ERM. Zur et al [8] reported the extent of the disorganization of the retinal inner layers (DRIL), a biomarker that can predict the vision change in patients with diabetic macular edema, had been associated with the visual prognosis after macular surgery for ERM.

OCTA has recently been used to evaluate ERM cases. Thus, Neris et al [11] reported that the foveal vessel density (VD) in eyes with an ERM was significantly higher than that of normal controls. They attributed this to a central displacement of the foveal capillaries. Kim et al [12] reported that the postoperative area of the FAZ was smaller in postoperative eyes with an ERM than the fellow eyes. Chen et al [13] compared the FAZ area and foveal VD before to that after vitrectomy for an ERM, and they reported an enlargement in FAZ area and a reduction in foveal VD. Kitagawa et al [14] also reported that the area of the FAZ increased significantly after vitrectomy for ERM, although the increase was small. Thus, evaluations of the retinal blood vessels of the inner retina by OCTA can be considered to be an evaluation of the structure of the inner retina.

OCTA can be used to evaluate not only the FAZ and VD but also the foveal morphology using the depth and morphologic information obtained at the same time as the structure. Samara et al. described a significant negative correlation between the FAZ area and CFT in normal eyes [15]. Our results confirmed the negative correlation between FAZ area and CFT even in ERM cases.

Although it was possible that the slow foveal structure changes after vitrectomy with ILM peeling, the mean FAZ area after vitrectomy was still significantly smaller at least 6 months follow-up than that of the control eyes. Interestingly, the FAZ area did not change significantly after vitrectomy although the CFT became thinner. These results suggest that the ERM affected the outer retina more than the inner retina. Because the postoperative BCVA was not significantly correlated with the FAZ area but was significantly correlated with the CFT, the morphological changes of the outer retina might affect the BCVA more than the changes of the inner retina.

In our cohort, there were six eyes that had a reduction of the FAZ area after the vitrectomy. Kumagai et al [16] observed a centripetal displacement of the foveal capillaries after ILM peeling for various macular diseases including an ERM. This suggests that the ILM peeling could be the cause of the smaller FAZ. Traction relief of centrifugal force after ILM peeling might be responsible for FAZ decrease. In eyes with an ERM, the enlargement or reduction of the FAZ area may depend on the degree of traction on the retina. In the 6 eyes with a reduction of FAZ area, the mean FAZ was larger than in the other eyes with the enlargement although the difference was not significant. For the CFT before the vitrectomy, there were significant differences in the eyes between the enlargement and reduction of the FAZ area after vitrectomy. Such cases were more likely to have a shorter duration of ERM, and the effects of ILM peeling may be greater than that of the ERM removal. Further studies with a larger number of cases are needed to prove the effect of ILM peeling on the reduction of the FAZ area.

We know that the dynamic changes of the foveal structures long periods after vitrectomy with ILM peeling [17]. Our results included only seven eyes with postoperative periods >6 months. However, the FAZ area was reduced in five of the seven eyes in spite of such cases having smaller FAZ areas compared to the normal control eyes. These results suggest that the area of the FAZ after surgery does not always enlarged even in the eyes with longer follow-up periods.

It is also important to evaluate the recovery of the foveal depression after vitrectomy. Our results showed a restoration of the foveal contour in about 60% of the eyes after the vitrectomy. About 80% of these eyes had an enlargement of the FAZ area. This suggests that the recovery of the foveal morphology including the FAZ area is associated with the restoration of foveal contour.

There are several limitations in this study including the small number of cases, especially the small number of vitrectomy cases. Another limitation was the short follow-up periods. In addition, the VD at the fovea was not evaluated to determine the degree of alterations of the capillaries. We also analyzed only the superficial capillary plexus because segmentation errors can occur in ERM eyes [18]. Thus, further studies are needed to evaluate the VD in the superficial and deep capillary plexus using the projection-resolved OCTA and other advanced devices.

In conclusion, our results demonstrate the importance of examining the FAZ area to evaluate the effects of an ERM on the structure and vasculature of the inner retina. The changes in the FAZ area after vitrectomy might be due to the release in the afferent retinal traction of the ERM and the centripetal displacement of the retina caused by the ILM peeling. We conclude that the traction of an ERM can alter the area of the FAZ and the thickness of the inner retina.

## Supporting information

S1 Table Baseline characteristics and changes before and after vitrectomy with ILM peeling for all eyes. (DOCX)
