# Supplementary material for: Foveal structure and vasculature in eyes with idiopathic epiretinal membrane
Source: PLoS One. 2019 Apr 2;14(4):e0214881. doi: 10.1371/journal.pone.0214881 (PMC6445520; doi:10.1371/journal.pone.0214881)
Supplement: S1 Table — (DOCX) [file pone.0214881.s001.docx]

| S1 Table. Baseline Characteristics and Changes before and after surgery | | | | |  |  |  |  |  |  |
| --- | --- | --- | --- | --- | --- | --- | --- | --- | --- | --- |
| No | Age | Gender | Surgery | pre/post | BCVA(Snellen) | FAZ | CFT | FAZ change | CFT change | F/U |
| 1 | 61 | F | + | pre | 1.2 | 0.142 | 301 | 31.7% | -37.1% | 338 |
|  |  |  |  | post | 1.2 | 0.187 | 189 |  |  |  |
| 2 | 81 | M | + | pre | 0.9 | 0.077 | 404 | 177.9% | -24.6% | 90 |
|  |  |  |  | post | 1.3 | 0.214 | 305 |  |  |  |
| 3 | 72 | M | + | pre | 0.3 | 0.038 | 423 | 236.8% | -50.5% | 127 |
|  |  |  |  | post | 0.8 | 0.128 | 210 |  |  |  |
| 4 | 60 | M | + | pre | 1.2 | 0.077 | 432 | 37.7% | -31.6% | 121 |
|  |  |  |  | post | 1.2 | 0.106 | 295 |  |  |  |
| 5 | 65 | F | + | pre | 1.0 | 0.084 | 366 | 47.6% | -21.0% | 27 |
|  |  |  |  | post | 0.9 | 0.124 | 289 |  |  |  |
| 6 | 59 | M | + | pre | 1.2 | 0.086 | 417 | 9.3% | -34.2% | 27 |
|  |  |  |  | post | 0.6 | 0.094 | 274 |  |  |  |
| 7 | 55 | M | + | pre | 0.8 | 0.426 | 280 | -72.1% | 19.7% | 366 |
|  |  |  |  | post | 1.2 | 0.119 | 335 |  |  |  |
| 8 | 60 | F | + | pre | 0.8 | 0.028 | 453 | 53.6% | -21.9% | 79 |
|  |  |  |  | post | 1.0 | 0.043 | 354 |  |  |  |
| 9 | 59 | F | + | pre | 1.2 | 0.097 | 384 | -45.4% | 6.4% | 443 |
|  |  |  |  | post | 1.2 | 0.053 | 409 |  |  |  |
| 10 | 68 | M | + | pre | 0.7 | 0.104 | 385 | -43.3% | -2.5% | 216 |
|  |  |  |  | post | 1.2 | 0.059 | 375 |  |  |  |
| 11 | 54 | M | + | pre | 1.2 | 0.218 | 343 | 2.3% | -32.8% | 23 |
|  |  |  |  | post | 1.0 | 0.223 | 231 |  |  |  |
| 12 | 49 | F | + | pre | 0.8 | 0.067 | 395 | 59.7% | -29.5% | 367 |
|  |  |  |  | post | 1.2 | 0.107 | 279 |  |  |  |
| 13 | 64 | M | + | pre | 1.2 | 0.076 | 359 | 21.1% | -21.6% | 34 |
|  |  |  |  | post | 1.3 | 0.092 | 282 |  |  |  |
| 14 | 64 | M | + | pre | 0.8 | 0.184 | 270 | -27.2% | -15.7% | 240 |
|  |  |  |  | post | 1.2 | 0.134 | 228 |  |  |  |
| 15 | 62 | F | + | pre | 1.0 | 0.143 | 252 | -22.4% | 10.9% | 177 |
|  |  |  |  | post | 1.2 | 0.111 | 280 |  |  |  |
| 16 | 63 | M | + | pre | 1.0 | 0.072 | 386 | 212.5% | -36.6% | 55 |
|  |  |  |  | post | 1.0 | 0.225 | 245 |  |  |  |
| 17 | 64 | M | + | pre | 1.2 | 0.073 | 328 | -20.5% | -16.3% | 100 |
|  |  |  |  | post | 0.7 | 0.058 | 275 |  |  |  |
| 18 | 78 | F | + | pre | 0.8 | 0.120 | 377 | 72.5% | -25.5% | 33 |
|  |  |  |  | post | 0.6 | 0.207 | 281 |  |  |  |
| 19 | 74 | F | + | pre | 0.6 | 0.072 | 405 | 105.6% | -33.3% | 29 |
|  |  |  |  | post | 1.2 | 0.148 | 270 |  |  |  |
| 20 | 72 | F | + | pre | 0.6 | 0.111 | 442 | 18.0% | -19.3% | 51 |
|  |  |  |  | post | 1.0 | 0.131 | 357 |  |  |  |
| 21 | 81 | M | - | - | 1.2 | 0.175 | 252 | - | | |
| 22 | 71 | M | - | - | 1.0 | 0.091 | 392 | - | | |
| 23 | 65 | F | - | - | 1.2 | 0.584 | 222 | - | | |
| 24 | 64 | F | - | - | 1.3 | 0.215 | 266 | - | | |
| 25 | 78 | M | - | - | 0.6 | 0.041 | 188 | - | | |
| 26 | 71 | F | - | - | 1.0 | 0.419 | 312 | - | | |
| 27 | 69 | M | - | - | 0.6 | 0.325 | 342 | - | | |
| 28 | 59 | F | - | - | 1.3 | 0.071 | 465 | - | | |
| 29 | 59 | F | - | - | 1.3 | 0.206 | 260 | - | | |
| 30 | 62 | M | - | - | 1.2 | 0.091 | 247 | - | | |
| 31 | 57 | F | - | - | 1.3 | 0.486 | 194 | - | | |
| 32 | 76 | F | - | - | 0.5 | 0.284 | 350 | - | | |
| 33 | 60 | F | - | - | 0.8 | 0.022 | 453 | - | | |
| 34 | 87 | M | - | - | 0.9 | 0.332 | 226 | - | | |
| 35 | 55 | M | - | - | 0.7 | 0.607 | 97 | - | | |
| 36 | 84 | M | - | - | 0.6 | 0.166 | 341 | - | | |
| 37 | 87 | M | - | - | 0.8 | 0.383 | 235 | - | | |
| 38 | 69 | F | - | - | 0.9 | 0.084 | 276 | - | | |
| 39 | 65 | F | - | - | 1.3 | 0.180 | 142 | - | | |
| 40 | 75 | F | - | - | 1.2 | 0.250 | 177 | - | | |
| 41 | 71 | M | - | - | 0.4 | 0.054 | 518 | - | | |
| 42 | 64 | F | - | - | 0.7 | 0.414 | 151 | - | | |
| 43 | 67 | M | - | - | 0.5 | 0.007 | 453 | - | | |
| 44 | 72 | M | - | - | 1.3 | 0.413 | 182 | - | | |
| 45 | 70 | F | - | - | 1.0 | 0.356 | 195 | - | | |
| 46 | 66 | M | - | - | 1.2 | 0.143 | 352 | - | | |
| 47 | 69 | F | - | - | 1.3 | 0.449 | 176 | - | | |
| 48 | 68 | M | - | - | 0.9 | 0.111 | 217 | - | | |
| 49 | 81 | M | - | - | 1.0 | 0.037 | 404 | - | | |
